# CircANKRD52 Augments the Growth and Invasion of Melanoma Cells by Sponging miR‐141‐3p and Upregulating PRKACB

**DOI:** 10.1111/jcmm.70909

**Published:** 2025-10-31

**Authors:** Shaojun Chu, Lingling Jia, Yulong Li, Changjiang Zhao, Yulin Sun, Qin Zhou, Dexiang Du, Zihan Li, Xin Huang, Hua Jiang, Baojin Wu, Yufei Li

**Affiliations:** ^1^ Department of Plastic Surgery, Shanghai East Hospital, School of Medicine Tongji University Shanghai China; ^2^ Department of Military Medical Psychology Air Force Medical University Xi'an China; ^3^ St Hugh's College University of Oxford Oxford UK; ^4^ Department of Dermatology, Hair Medical Center of Shanghai Tongji Hospital, Tongji Hospital, School of Medicine Tongji University Shanghai China; ^5^ Department of Plastic Surgery, Huashan Hospital Fudan University Shanghai China

**Keywords:** circANKRD52, growth, invasion, melanoma, miR‐141‐3p, PRKACB

## Abstract

Accumulating data have shown that circRNAs act pivotal roles in cancer progression. However, the role and molecular mechanism of circRNAs in melanoma remain undocumented. GEO dataset was used to identify the differentially expressed circRNAs between melanoma and normal tissues, and the expression and prognosis of circANKRD52 (hsa_circ_0026926) in melanoma were assessed by qRT‐PCR analysis. EdU, Transwell, Flow cytometry analysis, human umbilical vein endothelial cells (HUVECs) coculture assay as well as in vivo tumourigenesis models were utilised to assess cell growth and invasion. The specific binding between circANKRD52 and miR‐141‐3p was confirmed by bioinformatic analysis, RIP, RNA pull‐down, and luciferase reporter assays. The effect of circANKRD52 or miR‐141‐3p on PRKACB expression was evaluated by Western blot assay. We found that circANKRD52 was upregulated in melanoma tissue samples and cell lines and associated with poor survival and tumour recurrence in patients with melanoma. Knockdown of circANKRD52 repressed the growth and invasion of melanoma cells in vitro and in vivo, whereas ectopic expression of circANKRD52 promoted these effects. Moreover, circANKRD52 could bind to miR‐141‐3p, leading to upregulation of PRKACB, and downregulation of miR‐141‐3p restored melanoma cell growth and invasion. Our findings demonstrate that circANKRD52 promotes the growth and angiogenesis of melanoma cells by sponging miR‐141‐3p and upregulating PRKACB.

**Abbreviations**
ANKRD52: ankyrin repeat domain 52
CircRNA: circular RNA
CTLA4: cytotoxic T‐lymphocyte associated protein 4
DMEM: Dulbecco's modified Eagle's medium
EdU: 5‐ethynyl‐2′‐deoxyuridine
GAPDH: glyceraldehyde‐3‐phosphate dehydrogenase
HUVECs: human umbilical vein endothelial cells
miRNA: microRNA
MVD: micro vessel density
PRKACB: protein kinase cAMP‐activated catalytic subunit beta
RIP: RNA immunoprecipitation
VEGF: vascular endothelial growth factor

## 1 Introduction

Melanoma accounts for approximately 20% of the skin cancers, and its incidence and mortality increase year by year [1, 2]. Although systemic therapies have been applied for melanoma, such as targeted therapies using BRAF and MEK inhibitors or immunotherapies using anti‐PD‐1 and anti‐CTLA4, the treatment efficacy is temporary and unsatisfactory [3, 4]. Melanoma can produce resistance to clinical therapies and have poor prognosis due to its dissemination to distant sites [5]. Angiogenesis can maintain tumour growth and metastasis, leading to disease progression [6]. Therefore, identifying the underlying mechanisms involved in tumour progression is essential for the treatment of melanoma.

Circular RNAs (circRNAs) as a novel substyle of noncoding RNAs are characterised by a covalent close loop, specific back‐splicing sites, and high tissue specificity and conservation [7] and act critical roles in cancer progression including melanoma [8, 9, 10, 11]. It has been reported that circRNAs can act in cancer by sponging miRNAs [12, 13, 14], interacting with RNA‐binding proteins [15, 16, 17] and encoding proteins [18, 19, 20]. Likewise, circRNAs participate in the progression of melanoma [21]. They not only represent potential biomarkers for melanoma [11] but also act by regulating RNA‐binding proteins [22, 23, 24] and sponging miRNAs [25, 26]. Engineered circular RNAs (circRNAs) are emerging as promising platforms for RNA‐based vaccines in cancer treatment [27].

Previous study showed that circANKRD52 can enhance the tumourigenesis of hepatocellular carcinoma by sponging miR‐497‐5p and upregulating BIRC5 expression [28]. We herein identified another novel hsa_circ_0026926 derived from ANKRD52 and found that circANKRD52 (hsa_circ_0026926) promoted the growth and angiogenesis of melanoma cells by sponging miR‐141‐3p and might provide a potential biomarker for melanoma.

## 2 Materials and Methods

### 2.1 Sample Collection

Thirty‐two melanoma tissue samples were enrolled at Shanghai East Hospital from June 2012 to January 2019. A dataset GSE138734 was downloaded from the publicly accessible Gene Expression Omnibus (GEO) database (https://www.ncbi.nlm.nih.gov/geo/). The GSE138734 dataset comprised 6 primary melanoma (PM) and 6 normal skin (N) samples. All samples were hybridised with the HG‐U133 Plus 2.0 microarray (Affymetrix, Santa Clara, CA, USA). The present study was approved by the Ethics committee of Shanghai East Hospital (No. K‐KYSB‐2020‐0).

### 2.2 Cell Culture and Treatment

Human malignant melanoma cell lines A375, Hs294T, SK‐MEL‐28 were procured from American Type Culture Collection (ATCC, Manassas, VA, USA) and melanocytes were used as the control cells. The cells were cultured in Dulbecco's modified Eagle's medium (DMEM) with 10% foetal bovine serum (Gibco Company, Grand Island, NY, USA) at 37°C with 5% CO_2_ and 95% humidity. The cells were passaged when the cell confluence reached 90%. Overexpression (oe) vectors (oe‐circANKRD52 or oe‐PRKACB) and short hairpin RNA (shRNA) targeting circANKRD52 (sh‐circANKRD52, 5′‐ATCCTATTGCTCCTAGTCAGT‐3′) or sh‐PRKACB and the negative control vectors (oe‐NC or sh‐NC), the miR‐141‐3p mimic, miR‐141‐3p inhibitor, and the NC mimic and NC inhibitor were acquired from GenePharma (Shanghai, China). The vectors were transfected into the cells according to the instructions of a Lipofectamine 2000 kit (Thermo Fisher Scientific Inc., Waltham, MA, USA). After 48 h, stably transfected cells were collected for subsequent use.

### 2.3 Reverse Transcription Quantitative Polymerase Chain Reaction (RT‐qPCR)

Total RNA was extracted using the RNeasy Mini Kit (Qiagen, Valencia, CA, USA). The RNA sample was reversely transcribed to cDNA using an RT kit (RR047A, Takara Holdings Inc., Kyoto, Japan). Thereafter, real‐time qPCR was performed using an SYBR Premix EX Taq kit (RR420A, Takara) on a real‐time qPCR system (ABI7500, Applied Biosystems Inc., Carlsbad, CA, USA). All procedures were conducted in compliance with the manufacturer's instructions. GAPDH and U6 were used as the internal references for mRNAs and miRNA, respectively. Relative gene expression was quantified using the $2^{-∆∆C_{t}}$ method.

### 2.4 Western Blot Analysis

Total protein from cells was extracted using phenylmethylsulfonyl fluoride‐supplemented radio‐immunoprecipitation assay cell lysis buffer (C1055, Pulilai Gene Technology, Beijing, China). After protein concentration determination using a bicinchoninic acid kit (P0012‐1, Beyotime Biotechnology, Shanghai, China), an equal volume of protein (50 μg) was separated on 12% sodium dodecyl sulfate‐polyacrylamide gel electrophoresis and transferred onto polyvinylidene fluoride membranes. After being blocked in 5% non‐fat milk for 1 h, the membranes were hybridised with the primary antibodies against vascular endothelial growth factor (VEGF, sc‐7269, 1:1000, Santa, USA), PRKACB (XY12232, 1:2000, XYbscience), and GAPDH (ab9485, 1:2500, Abcam) at 4°C for 16 h. After that, the membranes were incubated with the secondary antibody goat anti‐rabbit IgG H&L (HRP) (ab97051, 1:2000, Abcam) at 37°C for 1 h. The protein blots were visualised using an enhanced chemiluminescence kit (Millipore, Corp. Billerica, MA, USA). Protein expression relative to GAPDH was examined using the Image J software.

### 2.5 5‐Ethynyl‐2′‐Deoxyuridine (EdU) and Transwell Assays

These assays were performed according to the previous report [14].

### 2.6 Fluorescence In Situ Hybridization (FISH)

The probe sequence for circANKRD52 (hsa_circ_0026926, 5′‐CAGCAATGAGCATGTGCTTT‐3′) was used to analyse the expression of circANKRD52 (red fluorescent signal) in melanoma cells. The detailed description of FISH analysis was executed as previously reported [14].

### 2.7 Actinomycin D and RNase R Treatment

Transcription was blocked by the addition of 2 mg/mL Actinomycin D. Total RNA was incubated for 30 min at 37°C with 3 U/μg of RNase R (Epicentre Technologies, USA).

### 2.8 RNA Immunoprecipitation (RIP) Assay

A Magna RIP kit (Millipore) was utilised for the RIP assay in strict accordance with the manufacturer's protocol. In brief, after being washed in phosphate‐buffered saline, cells were lysed in RIP lysis buffer and then centrifuged to collect the supernatant. The collected samples were incubated with 5 μg anti‐Ago2‐ (P10502500, Shenzhen Otwo Biotech Co. Ltd., Guangdong, China) or anti‐IgG (ab172730, Abcam)‐conjugated A/G agarose particles for RIP. The precipitated RNA was isolated using TRIzoL and purified, and the expression of circANKRD52 in the precipitated complexes was determined by RT‐qPCR.

### 2.9 RNA Pull‐Down Assay

Cells were transfected with 50 nm RNA biotin‐labelled NC and WT or Mut circANKRD52 probe. After 48 h, the cells were lysed in specific cell lysis buffer (Ambion, Austin, Texas, USA) for 10 min, and the cell lysates were sub‐packaged. The residual lysates were incubated with RNase‐free and tRNA‐precoated M‐280 streptavidin magnetic beads (Sigma‐Aldrich Chemical Company, St Louis, MO, USA) at 4°C for 3 h. After that, the samples were successively washed in cold lysis buffer, low‐salt buffer, and high‐salt buffer, and the miRNA was extracted for RT‐qPCR.

### 2.10 Dual Luciferase Reporter Gene Assay

The WT circANKRD52 and PRKACB luciferase vectors (circANKRD52‐WT and PRKACB‐WT) containing a putative binding site with miR‐141‐3p, and the corresponding MUT luciferase vectors (circANKRD52‐MUT and PRKACB‐MUT) were designed and provided by GenePharma. The WT and MUT luciferase vectors were co‐transfected with miR‐141‐3p mimic or NC mimic into 293T cells (ATCC). After 48 h, the luciferase activity in the cells was evaluated using a dual luciferase examination kit (D0010, Solarbio Science & Technology, Beijing, China), and the fluorescence intensity was examined using a GLomax20/20 Luminometer (E5311, Promega Corporation, Madison, Wisconsin, USA).

### 2.11 Flow Cytometry

An Annexin V‐fluorescein isothiocyanate (FITC) kit (BioVision, Milpitas, CA, USA) was used to examine apoptosis of cells. The cells were centrifuged, washed in phosphate‐buffered saline, and then incubated with 5 μL Annexin V‐FITC in the dark for 15 min and with 5 μL propidium iodide for 5 min. The apoptosis rate in cells was examined utilising flow cytometry (BD LSRFortessa, Franklin Lakes, NJ, USA) and analysed using Flow Jo 7.0 (FlowJo LLC, USA).

### 2.12 Tube Formation Assay

Human umbilical vein endothelial cells (HUVECs, INS‐1, Zishi Biotechnology Co. Ltd., Shanghai, China) were routinely cultured and collected. The culture medium of A375 and SK‐MEL‐28 cells was collected, centrifuged at 3000 r/min for 3 min to collect the supernatant, termed conditioned medium. The HUVECs were resuspended in the conditioned medium to 1 × 10^5^ cells/mL. Then, 500 μL cell suspension was loaded in Matrigel‐precoated 24‐well plates and cultured at 37°C with 5% CO_2_. After 24 h, the number of tubes formed by HUVECs in different conditioned media was observed under an inverted microscope with five random fields included.

### 2.13 Animal Experiments

Thirty BALB/c nude mice (4 weeks old, 18–25 g) acquired from the Experimental Animal Center of Sun Yat‐sen University (Guangdong, China) were randomly allocated into two groups. SK‐MEL‐28 cells (1 × 10^6^) transfected with sh‐NC (*n* = 5) or sh‐circANKRD52 (*n* = 5) and oe‐NC (*n* = 5) or oe‐circANKRD52 (*n* = 5) were subcutaneously injected into the nude mice at the abdomen. The volume (*V*) of xenograft tumours was evaluated every 5 days as follows: *V* = 1/2 × length × width^2^. After 20 days, the mice were euthanized via intraperitoneal injection of 1% pentobarbital sodium. Then, the tumours were taken out for weighing and histological examination. The animal study was approved by the Ethics Committee of Shanghai East Hospital (No. K‐KYSB‐2020‐0).

### 2.14 Immunohistochemical (IHC) Staining

Tumour tissue samples were cut into 4‐μm sections, dewaxed, rehydrated, soaked in 3% H_2_O_2_ for 10 min, and underwent high‐pressure treatment for 90 s of antigen retrieval. The sections were blocked in 5% bovine serum albumin at 37°C for 30 min and then incubated with 50 μL anti‐CD31 (ab28364, 1: 100, Abcam) and anti‐vascular endothelial growth factor (VEGF, sc‐7269, 1:1000, Santa, USA) at 4°C overnight. After that, the sections were incubated with 50 μL HRP‐conjugated anti‐IgG (ab205718, 1:100 Abcam) at 37°C for 30 min. After colour development by 3,3′‐diaminobenzidine, the sections were counter‐stained with haematoxylin. The staining was observed under the microscope and scored as previously reported (14). An over 25% staining rate (pale brown) in cytoplasm, cell membrane or vascular endothelium was deemed as positive staining. The micro vessel density (MVD) was evaluated according to the positive staining of CD31. A brownish endothelial cell cluster or a single endothelial cell showing clear boundaries with tumour cells, micro vessels and the surrounding connective tissues were counted as a micro vessel.

### 2.15 Statistical Analysis

SPSS system (version 21.0, IBM Corp. Armonk, NY, USA) was used for data analysis. Data were expressed as mean ± standard deviation (SD) from no less than three independent experiments. Differences were compared by unpaired *t* test, or the one‐ or two‐way analysis of variance (ANOVA). Correlations between variables were determined by Pearson's correlation analysis. *p* < 0.05 was considered to show a statistically significant difference.

## 3 Results

### 3.1 CircANKRD52 Is Highly Expressed in Melanoma Tissues and Associated With Poor Prognosis in Patients With Melanoma

**FIGURE 1:**
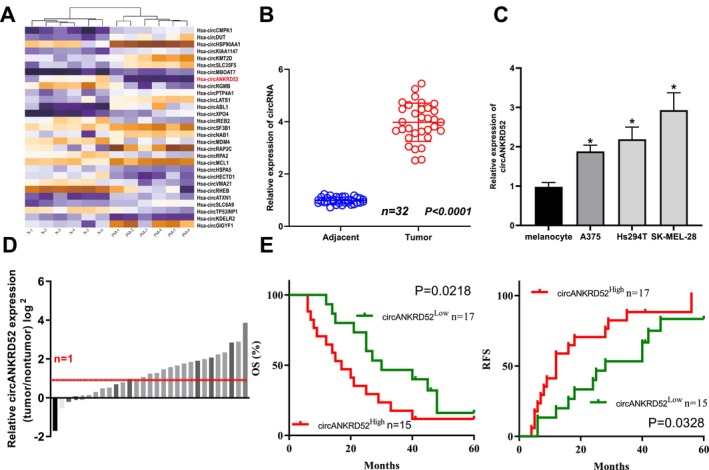
CircANKRD52 is upregulated and associated with poor prognosis in patients with melanoma. (A) Heatmap analysis of the differentially expressed circRNAs between melanoma and normal tissues (*n* = 6). (B) RT‐qPCR analysis of the expression levels of circANKRD52 in 32 pairs of melanoma and normal tissues. (C) RT‐qPCR analysis of the expression levels of circANKRD52 in melanoma cell lines. (D) The melanoma patients were divided into circANKRD52‐high (*n* = 17) and circANKRD52‐low groups (*n* = 15). (E) Kaplan–Meier analysis of the association of circANKRD52 expression with overall survival and tumour recurrence in patients with melanoma. Data shown are the mean ± SEM of three experiments. *p < 0.05.

A GSE138734 dataset was used to assess the differential expression of circRNAs in melanoma tissues. Using |log2FC| > 2 and *p* < 0.01, 29 differentially expressed circRNAs between the primary melanoma (PM) and normal skin (N) were identified, of which 14 circRNAs were upregulated and 15 were downregulated (Figure 1A). Among these upregulated circRNAs, hsa_circ_0026926 (circANKRD52) had the most significant upregulation and was further validated by RT‐qPCR in 32 melanoma tissues as compared with the normal tissues (Figure 1B). The expression of circANKRD52 was also increased in A375, Hs294T, and SK‐MEL‐28 cell lines as compared with melanocytes (Figure 1C). According to the circANKRD52 expression levels, survival time, and survival status, we obtained the cutoff value of circANKRD52 in melanoma and divided the patients into high‐circANKRD52 and low‐circANKRD52 groups (Figure 1D). The Pearson's chi‐square test was applied to examine the association between circANKRD52 expression and the clinical characteristics in patients with melanoma. We found that high expression of circANKRD52 was correlated with the Breslow thickness and the Clark levels but had no association with the age or sex of patients (Table 1). Kaplan–Meier analysis uncovered that the patients with circANKRD52‐high expression harboured poorer survival and higher tumour recurrence as compared with those with circANKRD52‐low expression (Figure 1E).

**TABLE 1:** Correlation between circANKRD52 expression and the clinicopathological parameters of patients with melanoma.

Variable	circANKRD52		*p*
	High (*n* = 17)	Low (*n* = 15)	
Age (years)			
≤ 63	5	7	0.417
> 63	12	8	
Sex			
Male	9	6	0.672
Female	8	9	
Breslow thickness			
≤ 0.75 mm	8	13	0.029
> 0.75 mm	9	2	
Clark level			
I–III	3	8	0.017
IV–V	14	7	
Ulceration			
Present	9	10	0.145
Absent	8	5	

*Note:* Data shown are the mean ± SEM of three experiments. *p < 0.05.

### 3.2 Identification of the Characteristics of circANKRD52 in Melanoma Cells

**FIGURE 2:**
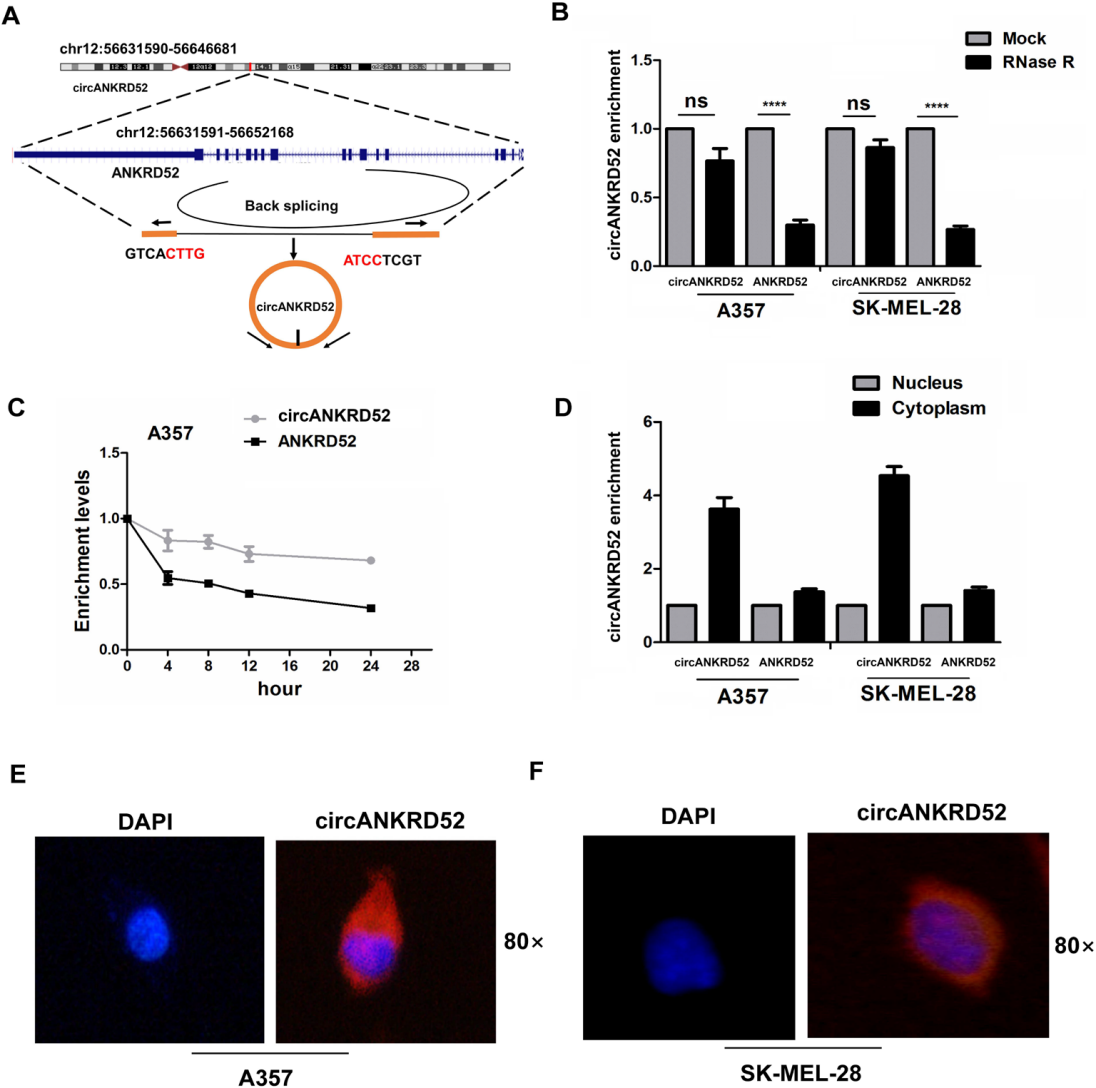
Identification of a circANKRD52 in melanoma cells. (A) The genomic loci of circANKRD52. (B) RT‐qPCR analysis of the expression levels of circANKRD52 and ANKRD52 after treatment with RNase R in A375 and SK‐MEL‐28 cells. (C) RT‐qPCR analysis of the transcriptional stability of circANKRD52 and ANKRD52 after treatment with Actinomycin D in A375 cells. (D) RT‐qPCR analysis of the location of circANKRD52 in A375 and SK‐MEL‐28 cells. (E) FISH analysis of the location of circANKRD52 in A375 and SK‐MEL‐28 cells. Data shown are the mean ± SEM of three experiments. ****p < 0.0001.

According to the annotation from Circular RNA Interactome (https://circinteractome.nia.nih.gov/index.html), hsa_circ_0026926 (chr12:56631590–56646881) is derived from exon 11, 28 regions within the ANKRD52 locus and is termed circANKRD52 (Figure 2A). In addition, after exposure to RNase R treatment, we measured the expression levels of circANKRD52 in A375 and SK‐MEL‐28 cell lines and found that circANKRD52 exerted resistance to RNase R treatment as compared with linear ANKRD52 (Figure 2B). After A375 cells were exposed to Actinomycin D treatment for 12 h, circANKRD52 produced more obvious stability than its linear ANKRD52 (Figure 2C). Cell localisation analysis showed that circANKRD52 was predominantly localised in the cytoplasm of A375 and SK‐MEL‐28 cells (Figure 2D–F).

### 3.3 CircANKRD52 Augments the Proliferation and Invasion of Melanoma Cells

**FIGURE 3:**
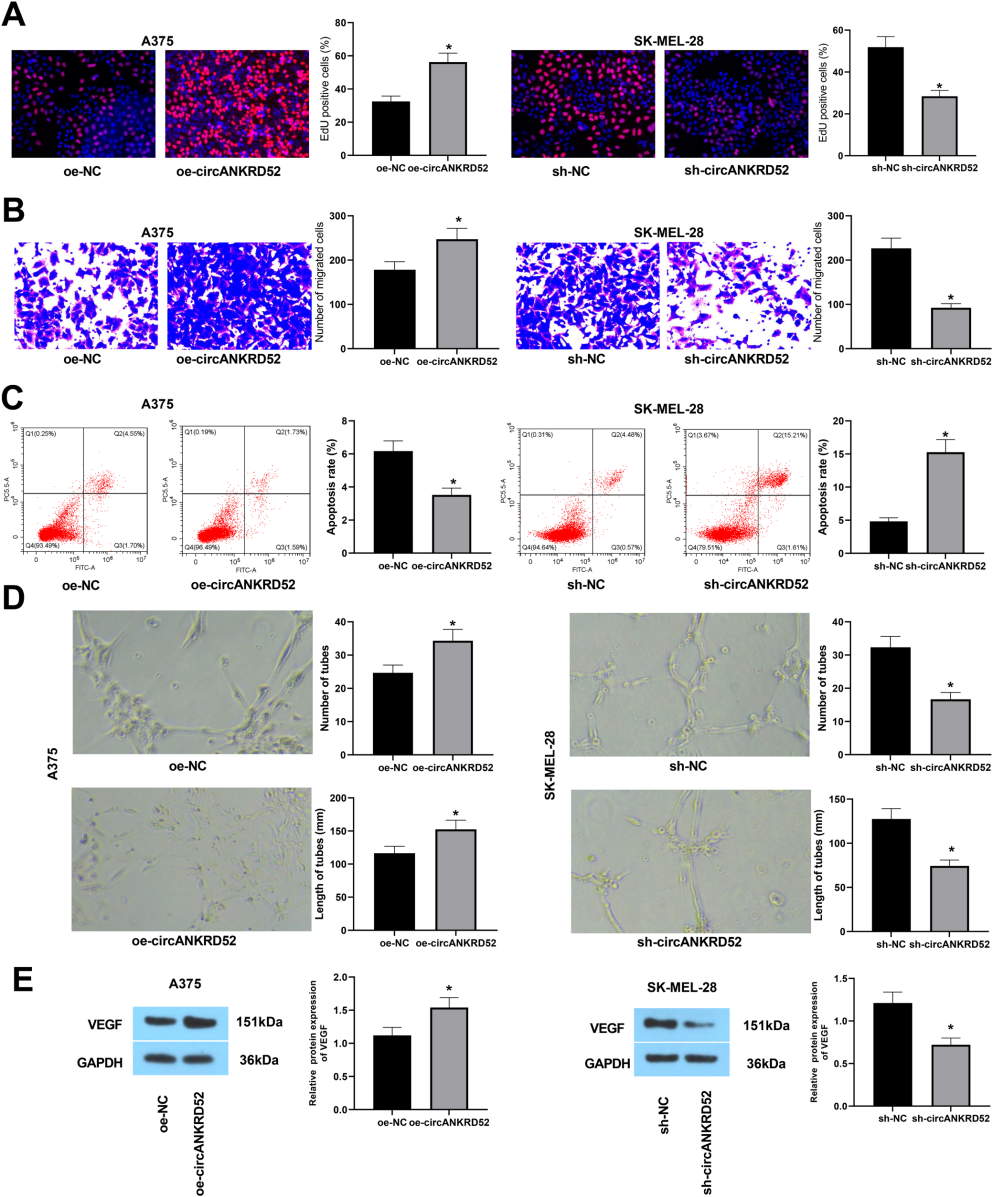
CircANKRD52 augments proliferation and invasion of melanoma cells. (A) EdU analysis of the cell proliferation activity after transfection with oe‐circANKRD52 into A375 cells or sh‐circANKRD52 into SK‐MEL‐28 cells. (B) Transwell analysis of the cell invasion capabilities after transfection with oe‐circANKRD52 into A375 cells or sh‐circANKRD52 into SK‐MEL‐28 cells. (C) Flow cytometry analysis of the cell apoptosis after transfection with oe‐circANKRD52 into A375 cells or sh‐circANKRD52 into SK‐MEL‐28 cells. (D) Tube formation assay analysis of the angiogenesis ability after transfection with oe‐circANKRD52 into A375 cells or sh‐circANKRD52 into SK‐MEL‐28 cells. (E) Western blot analysis of the protein levels of VEGF after transfection with oe‐circANKRD52 into A375 cells or sh‐circANKRD52 into SK‐MEL‐28 cells. Data shown are the mean ± SEM of three experiments. *p < 0.05.

Gain‐ and loss‐of‐functions were used to evaluate the role of circANKRD52 in melanoma cells. The transfections of oe‐circANKRD52 or sh‐circANKRD52 in A375 or SK‐MEL‐28 cells, respectively, were confirmed by RT‐qPCR (Figure S1). The EdU assay suggested that the proliferation was increased after circANKRD52 overexpression in A375 cells but decreased after circANKRD52 knockdown in SK‐MEL‐28 cells (Figure 3A). The Transwell assay showed that the invasive abilities of A375 cells were augmented by oe‐circANKRD52 but weakened by sh‐circANKRD52 in SK‐MEL‐28 cells (Figure 3B). However, the apoptosis of A375 cells, indicated by flow cytometry analysis, was reduced after circANKRD52 overexpression but elevated by sh‐circANKRD52 in SK‐MEL‐28 cells (Figure 3C).

The role of circANKRD52 in angiogenesis of melanoma cells was further examined. HUVECs were cultured in the conditioned medium of A375 cells or SK‐MEL‐28 cells. We found that the angiogenesis ability of HUVECs was increased by circANKRD52‐overexpressing medium in A375 cells but lowered by circANKRD52‐silencing medium in SK‐MEL‐28 cells (Figure 3D). In addition, Western blot analysis showed that the expression of angiogenesis‐related protein VEGF was significantly increased in circANKRD52‐overexpressed A375 cells while reduced in circANKRD52‐silencing SK‐MEL‐28 cells (Figure 3E).

### 3.4 CircANKRD52 Acts as a Sponge of miR‐141‐3p in Melanoma Cells

**FIGURE 4:**
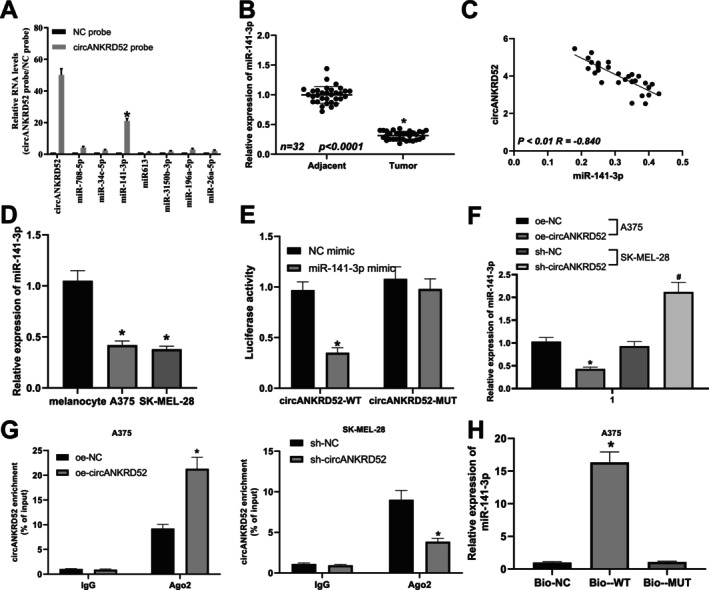
The circANKRD52 acts as a sponge of miR‐141‐3p in melanoma cells. (A) The RNA pull‐down analysis of the binding between circANKRD52 probe and miRNA in A375 cells. (B) RT‐qPCR analysis of the expression levels of miR‐141‐3p in melanoma and the adjacent normal tissues. (C) Pearson's correlation analysis of the correlation between miR‐141‐3p and circANKRD52 in melanoma tissues. (D) RT‐qPCR analysis of the expression of miR‐141‐3p in melanoma cell lines (A375, Hs294T, and SK‐MEL‐28). (E) The dual luciferase reporter assay indicated the binding between circANKRD52 3′UTR and miR‐141‐3p. (F) RT‐qPCR analysis of the expression of miR‐141‐3p after transfection with oe‐circANKRD52 into A375 cells or sh‐circANKRD52 into SK‐MEL‐28 cells. (G) RIP analysis of the binding ability between circANKRD52 and Ago2 after transfection with oe‐circANKRD52 into A375 cells or sh‐circANKRD52 into SK‐MEL‐28 cells. (H) RNA pull‐down assay analysis of the binding between Bio‐circANKRD52 WT and miR‐141‐3p. Data shown are the mean ± SEM of three experiments. *p < 0.05.

To confirm circANKRD52‐specific binding with miRNAs, we identified 8 miRNAs which could be bound by circANKRD52 by using starBaseV2.0 (https://starbase.sysu.edu.cn/starbase2/index.php). Then, we used a circANKRD52 probe to carry out an RNA in vivo precipitation assay. The circANKRD52‐specific probes were used to purify the RNA pulled down from circANKRD52, and RT‐qPCR showed that the enrichment levels of miR‐141‐3p rather than the other miRNAs were significantly increased compared with controls in A375 cells (Figure 4A). The putative binding site between circANKRD52 and miR‐141‐3p could be indicated in Figure S2A. The expression of miR‐141‐3p was found reduced (Figure 4B) and harboured an inverse correlation with the circANKRD52 expression in melanoma tissues (Figure 4C). The expression of miR‐141‐3p was decreased in A375 and SK‐MEL‐28 cells relative to melanocytes (Figure 4D). To examine whether circANKRD52 can bind with miR‐141‐3p, the circANKRD52‐WT and circANKRD52‐MUT luciferase vectors were constructed and co‐transfected with miR‐141‐3p mimic into 293T cells. It was found that the luciferase activity of the circANKRD52‐WT vector was significantly reduced by miR‐141‐3p mimic, but that was unaffected by circANKRD52‐MUT or NC mimic (Figure 4E). The RT‐qPCR results suggested that the expression of miR‐141‐3p was reduced in the circANKRD52‐overexpressing A375 cells but elevated in the circANKRD52‐silencing SK‐MEL‐28 cells (Figure 4F). Moreover, a RIP assay was performed to validate the binding between circANKRD52 and Ago2‐miR‐141‐3p complex in melanoma cells, and we found that overexpression of circANKRD52 promoted the binding between circANKRD52 and Ago2 in the immunoprecipitates but silencing of circANKRD52 reduced their binding abilities (Figure 4G). Likewise, the RNA pull‐down assay showed that abundant miR‐141‐3p was detected in the melanoma cells pulled down by circANKRD52‐WT (Figure 4H and Figure S2B).

### 3.5 miR‐141‐3p Reverses circANKRD52‐Induced Melanoma Cell Proliferation and Invasion

**FIGURE 5:**
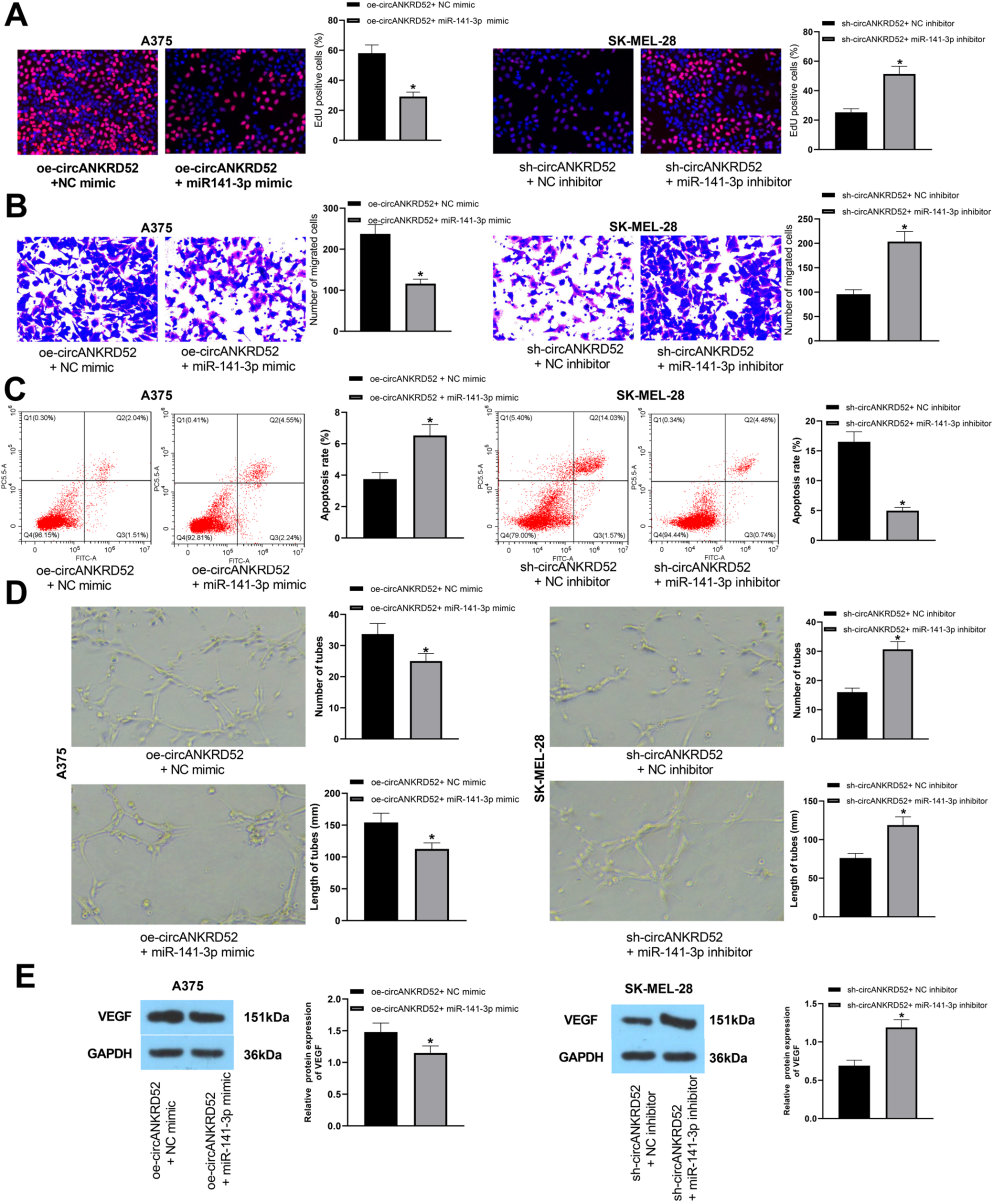
The miR‐141‐3p reverses circANKRD52‐induced melanoma cell proliferation and invasion. (A) EdU analysis of the cell proliferation activity after co‐transfection with oe‐circANKRD52 and miR‐141‐3p mimic into A375 cells or sh‐circANKRD52 and miR‐141‐3p inhibitor into SK‐MEL‐28 cells. (B) Transwell analysis of the cell invasion capabilities after co‐transfection with oe‐circANKRD52 and miR‐141‐3p mimic into A375 cells or sh‐circANKRD52 and miR‐141‐3p inhibitor into SK‐MEL‐28 cells. (C) Flow cytometry analysis of the cell apoptosis after co‐transfection with oe‐circANKRD52 and miR‐141‐3p mimic into A375 cells or sh‐circANKRD52 and miR‐141‐3p inhibitor into SK‐MEL‐28 cells. (D) Tube formation assay analysis of the angiogenesis ability after co‐transfection with oe‐circANKRD52 and miR‐141‐3p mimic into A375 cells or sh‐circANKRD52 and miR‐141‐3p inhibitor into SK‐MEL‐28 cells. (E) Western blot analysis of the protein levels of VEGF after co‐transfection with oe‐circANKRD52 and miR‐141‐3p mimic into A375 cells or sh‐circANKRD52 and miR‐141‐3p inhibitor into SK‐MEL‐28 cells. Data shown are the mean ± SEM of three experiments. *p < 0.05.

To explore the potential involvement of miR‐141‐3p in circANKRD52‐mediated events, miR‐141‐3p mimic was further transfected into A375 cells while miR‐141‐3p inhibitor was transfected into SK‐MEL‐28 cells after oe‐circANKRD52 and sh‐circANKRD52 transfections. The transfections of miR‐141‐3p mimic or miR‐141‐3p inhibitor were confirmed by RT‐qPCR (Figure S3). We found that miR‐141‐3p mimic reversed circANKRD52‐induced cell proliferation and invasion in A375 cells, whereas miR‐141‐3p inhibitor counteracted sh‐circANKRD52‐caused antitumour effects in SK‐MEL‐28 cells (Figure 5A,B). However, flow cytometry analysis showed that miR‐141‐3p mimic reversed circANKRD52‐induced apoptosis decrease in A375 cells, whereas miR‐141‐3p inhibitor attenuated sh‐circANKRD52‐led apoptosis increase in SK‐MEL‐28 cells (Figure 5C). In addition, miR‐141‐3p mimic reversed circANKRD52‐induced increased HUVECs tube formation in A375 cells, whereas miR‐141‐3p inhibitor attenuated sh‐circANKRD52‐induced decreased HUVECs tube formation in SK‐MEL‐28 cells (Figure 5D). Western blot analysis showed miR‐141‐3p mimic reversed circANKRD52‐induced VEGF upregulation in A375 cells, whereas miR‐141‐3p inhibitor attenuated sh‐circANKRD52‐induced VEGF downregulation in SK‐MEL‐28 cells (Figure 5E).

### 3.6 PRKACB Is a Direct Target of miR‐141‐3p in Melanoma Cells

**FIGURE 6:**
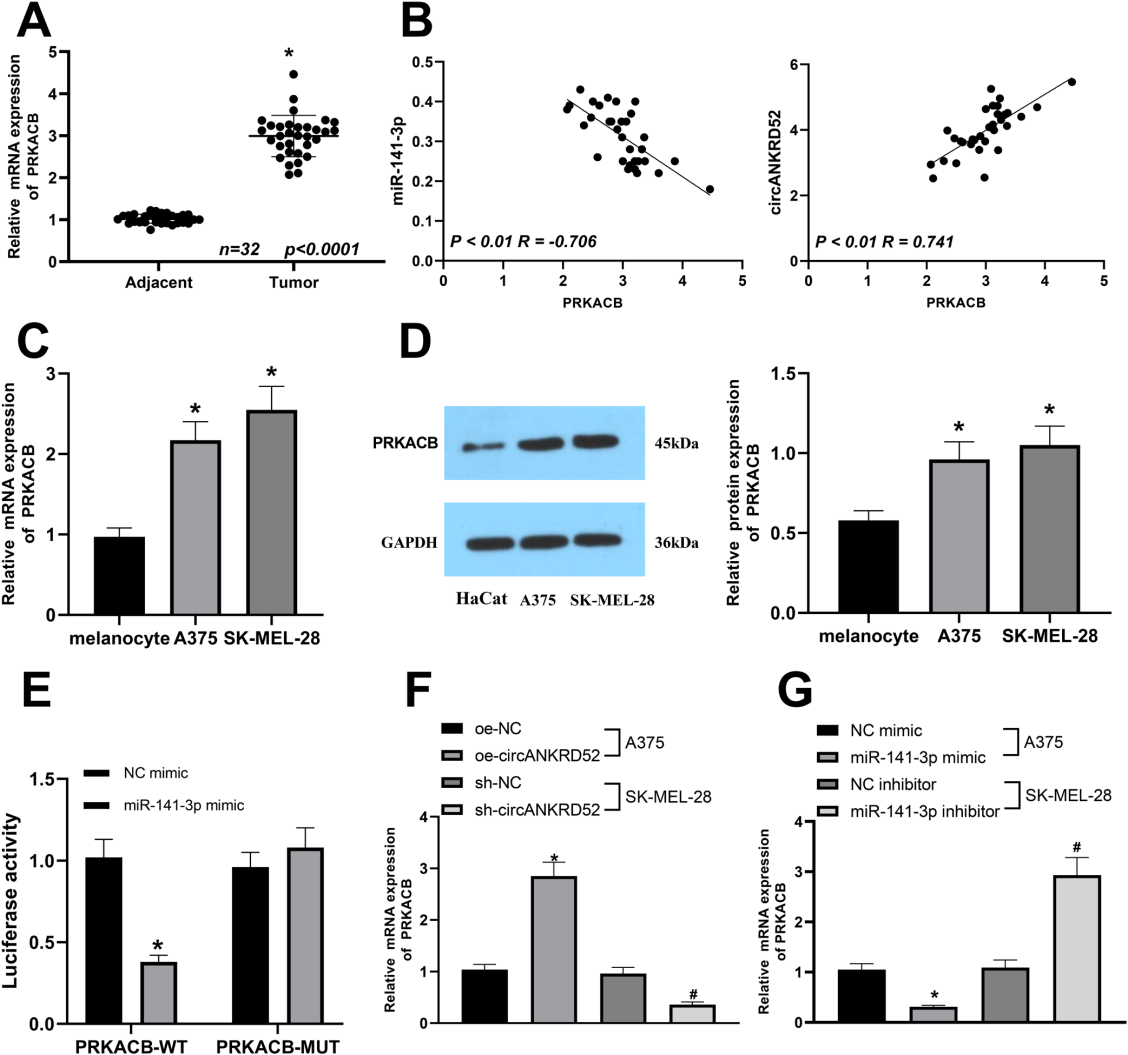
PRKACB is identified as a direct target of miR‐141‐3p in melanoma cells. (A) RT‐qPCR analysis of the PRKACB expression in melanoma and the adjacent normal tissues. (B) Pearson's correlation analysis of the correlations of PRKACB with miR‐141‐3p or circANKRD52 expression in melanoma tissues. (C, D) RT‐qPCR and Western blot analysis of the expression of PRKACB in A375, SK‐MEL‐28, and melanocytes. (E) The dual luciferase reporter assay indicated the binding between miR‐141‐3p and PRKACB 3'UTR. (F) RT‐qPCR analysis of the expression of PRKACB after transfection with oe‐circANKRD52 into A375 cells or sh‐circANKRD52 into SK‐MEL‐28 cells. (G) RT‐qPCR analysis of the expression of PRKACB after transfection with miR‐141‐3p mimic into A375 cells or miR‐141‐3p inhibitor into SK‐MEL‐28 cells. Data shown are the mean ± SEM of three experiments. *p < 0.05. #*p*  < 0.05.

To identify the target genes of miR‐141‐3p, we screened an oncogenic PRKACB as the possible target of miR‐141‐3p by using starBaseV2.0 (https://starbase.sysu.edu.cn/starbase2/index.php). The increased expression of PRKACB in melanoma tissues was determined by RT‐qPCR (Figure 6A), and PRKACB possessed an inverse correlation with miR‐141‐3p expression but a positive correlation with circANKRD52 expression (Figure 6B). PRKACB was validated to be highly expressed in A375 and SK‐MEL‐28 cells compared to melanocytes (Figure 6C,D). To validate the binding between miR‐141‐3p and PRKACB, the PRKACB‐WT and PRKACB‐MUT luciferase vectors were constructed and co‐transfected with miR‐141‐3p mimic or NC mimic into 293T cells. It was found that the luciferase activity in the cells co‐transfected with miR‐141‐3p mimic and PRKACB‐WT was reduced, while the activity in the cells transfected with NC mimic or PRKACB‐MUT was unchanged (Figure 6E). RT‐qPCR results showed that the expression of PRKACB was increased by oe‐circ_0026939 or miR‐141‐3p inhibitor but reduced by sh‐circANKRD52 or miR‐141‐3p mimic (Figure 6F,G).

### 3.7 PRKACB Mediates circANKRD52 to Promote Melanoma Cell Proliferation and Invasion

**FIGURE 7:**
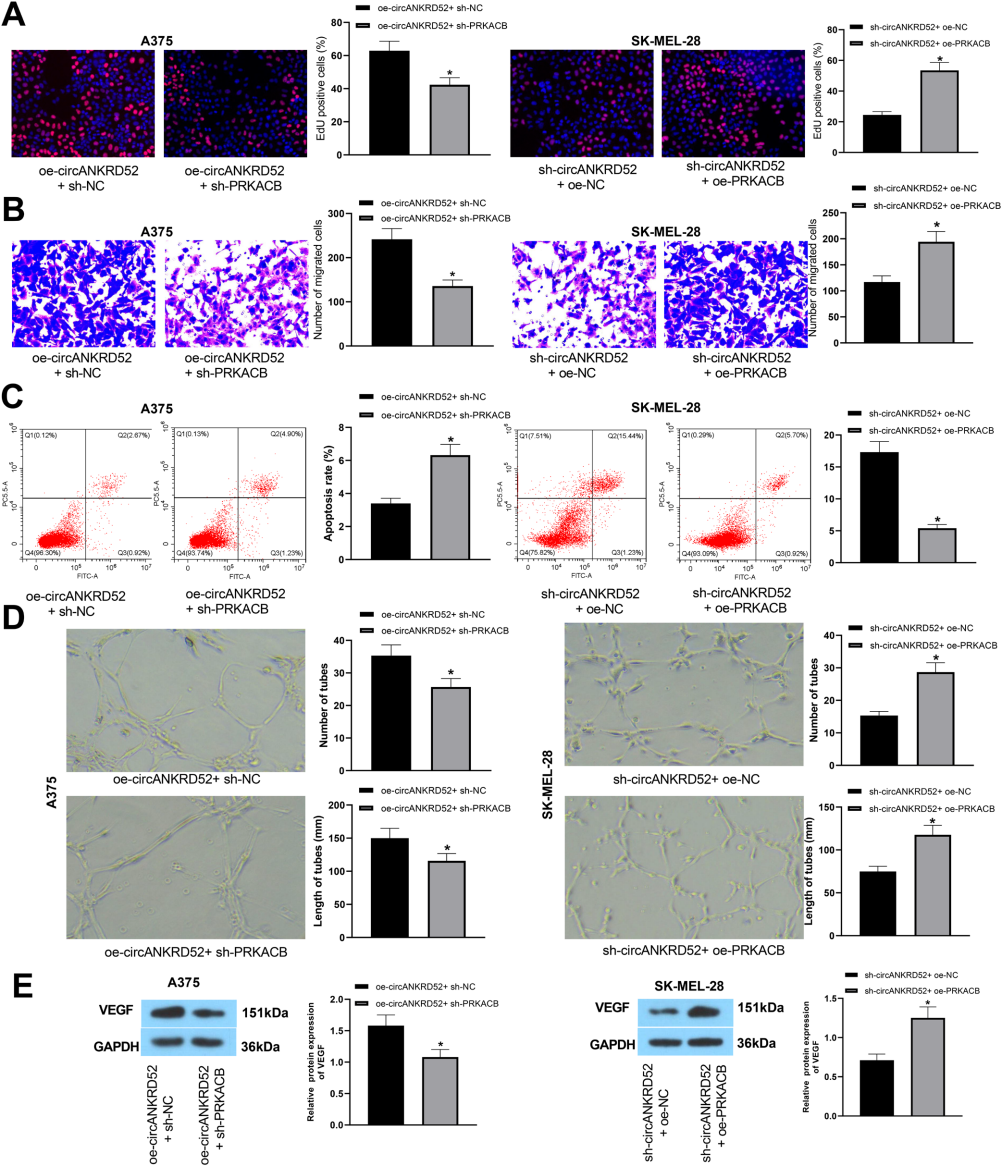
The circANKRD52/PRKACB axis mediates melanoma cell proliferation and invasion. (A) EdU analysis of the cell proliferation activity after co‐transfection with oe‐circANKRD52 and sh‐PRKACB into A375 cells or sh‐circANKRD52 and oe‐PRKACB into SK‐MEL‐28 cells. (B) Transwell analysis of the cell invasion capabilities after co‐transfection with oe‐circANKRD52 and sh‐PRKACB into A375 cells or sh‐circANKRD52 and oe‐PRKACB into SK‐MEL‐28 cells. (C) Flow cytometry analysis of the cell apoptosis after co‐transfection with oe‐circANKRD52 and sh‐PRKACB into A375 cells or sh‐circANKRD52 and oe‐PRKACB into SK‐MEL‐28 cells. (D) Tube formation assay analysis of the angiogenesis ability after co‐transfection with oe‐circANKRD52 and sh‐PRKACB into A375 cells or sh‐circANKRD52 and oe‐PRKACB into SK‐MEL‐28 cells. (E) Western blot analysis of the protein levels of VEGF after co‐transfection with oe‐circANKRD52 and sh‐PRKACB into A375 cells or sh‐circANKRD52 and oe‐PRKACB into SK‐MEL‐28 cells. Data shown are the mean ± SEM of three experiments. *p < 0.05.

To validate the potential role of the circANKRD52/PRKACB axis in melanoma, sh‐PRKACB was further introduced in the oe‐circANKRD52‐transfected A375 cells, or oe‐PRKACB was introduced in the sh‐circANKRD52‐transfected SK‐MEL‐28 cells, and the transfections were confirmed by RT‐qPCR (Figure S4). It was found that the proliferation and invasion of A375 cells enhanced by oe‐circANKRD52 were abolished by sh‐PRKACB, while those reduced by sh‐circANKRD52 were reversed by oe‐PRKACB in SK‐MEL‐28 cells (Figure 7A,B). The apoptosis of A375 cells inhibited by oe‐circANKRD52 was reversed by sh‐PRKACB, while this increased by sh‐circANKRD529 was reversed by oe‐PRKACB in SK‐MEL‐28 cells (Figure 7C). In addition, the tube formation of HUVECs increased by oe‐circANKRD52 was reversed by sh‐PRKACB, while this decreased by sh‐circ_0026939 was reversed by oe‐PRKACB in SK‐MEL‐28 cells (Figure 7D). The protein level of VEGF was increased by oe‐circANKRD52 and was reversed by sh‐PRKACB, while this decreased by sh‐circANKRD52 was attenuated by oe‐PRKACB in SK‐MEL‐28 cells (Figure 7E).

### 3.8 The circANKRD52 Promotes In Vivo the Tumourigenesis of Melanoma Cells

**FIGURE 8:**
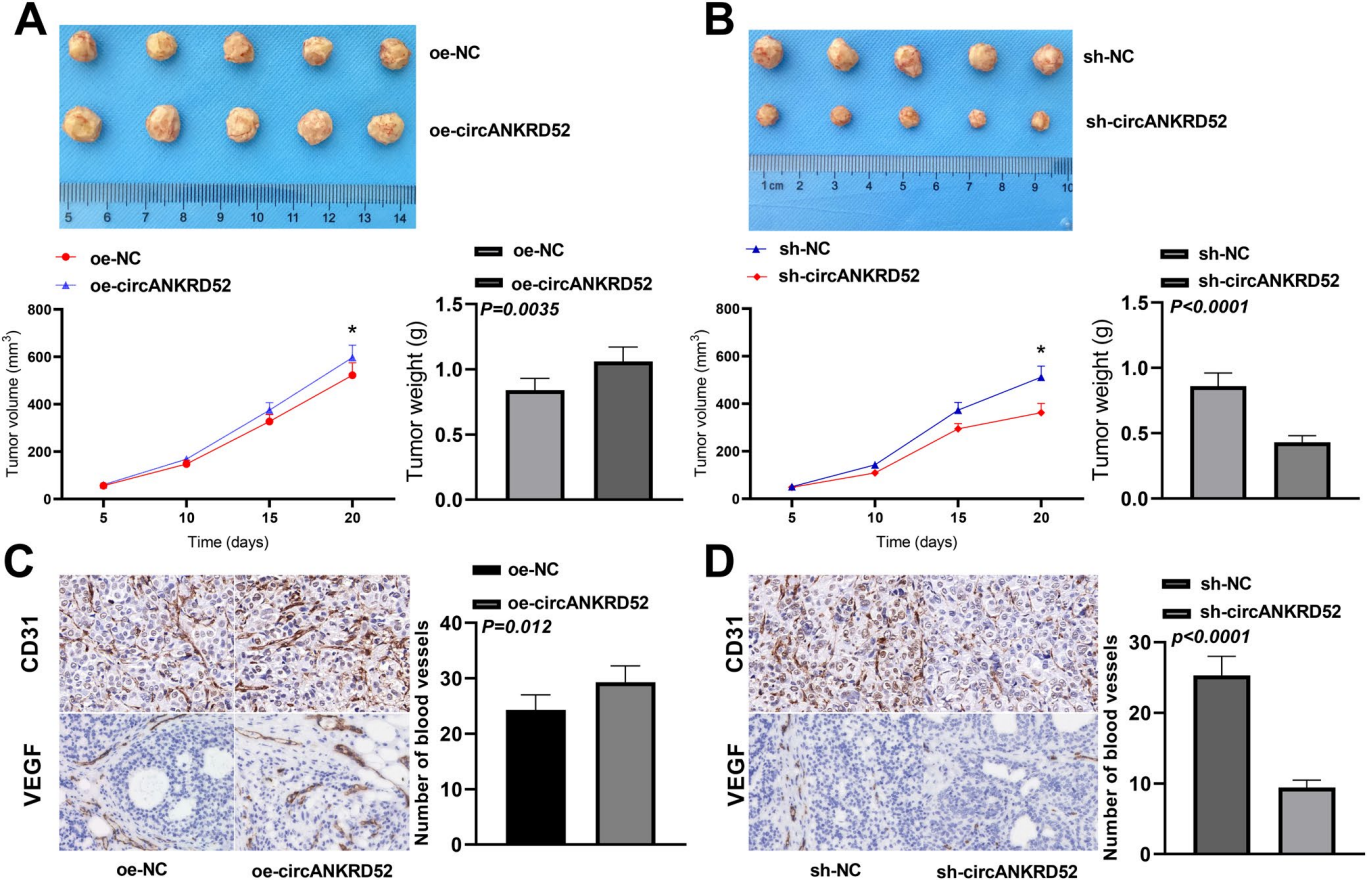
The circANKRD52 promotes in vivo the tumorigenesis of melanoma cells. (A) The effects of circANKRD52 overexpression on the xenograft tumour growth and weight in mice. (B) The effects of circANKRD52 knockdown on the xenograft tumour growth and weight in mice. (C) IHC analysis of the effects of circANKRD52 overexpression on CD31 and VEGF expression in xenograft tumour tissues. (D) IHC analysis of the effects of circANKRD52 knockdown on CD31 and VEGF expression in xenograft tumour tissues. *N* = 5 in each group.

To validate the role of circANKRD52 in melanoma cells, the animal experiments were performed. SK‐MEL‐28 cells stably transfected with oe‐NC (*n* = 5)/oe‐circ_0026939 (*n* = 5) or sh‐NC (*n* = 5)/sh‐circANKRD52 (*n* = 5) were implanted into mice. It was found that overexpression of circANKRD52 promoted tumour growth and increased the tumour weight in mice (Figure 8A), but downregulation of circANKRD52 repressed the tumour growth and lowered the tumour weight in mice as compared with the control group (Figure 8B). IHC staining was performed to examine the CD31 and VEGF expression in tumour tissues of mice. We found that the positive staining of CD31 and VEGF was increased by overexpression of circANKRD52 (Figure 8C), but decreased by downregulation of circANKRD52 as compared with the control group (Figure 8D).

## 4 Discussion

The prognosis of the early‐diagnosed melanoma cases is relatively favourable after surgical resection, but those at the late stages have poor outcomes [29]. Dissemination of melanomas to lymphatic vessels surrounding the tumour tissues is the crucial step in distant metastasis [6]. Our findings herein demonstrated that circANKRD52 was upregulated in melanoma tissues and associated with poor prognosis in patients with melanoma. The circANKRD52 promoted the growth and invasion of melanoma cells by sponging miR‐141‐3p and upregulating PRKACB, and might provide a potential biomarker for treatment of melanoma.

Increasing data unveil that deregulated circRNAs are involved in the tumourigenesis and metastasis of melanoma [30, 31]. The expression of circ_0082835 [31], circ_0062270 [32], circ_0079593 [33], circ_0020710 [26], circ_0025039 [34] and circ_0002770 [35] has been reported to be increased while circZNF609 expression is decreased in melanoma tissues [30]. Herein, we identified a novel hsa_circ_0026926 derived from ANKRD52 and found circANKRD52 was upregulated in melanoma tissues and associated with the Breslow thickness, Clark levels, and poor prognosis in patients with melanoma, indicating that circANKRD52 might be a promising prognostic factor for melanoma patients.

Accumulating evidence shows that circRNAs can act as oncogenes or tumour suppressors in melanoma. For example, circ_0020710, circ_0025039, and circ_0002770 facilitate tumour progression and immune evasion in melanoma [26, 34, 35], whereas circZNF609 represses tumour metastasis in melanoma [30]. Herein, we found that the knockdown of circANKRD52 repressed the growth and invasion of melanoma cells in vitro and in vivo, whereas the ectopic expression of circANKRD52 promoted these effects, suggesting that circANKRD52 might be a tumour‐promoting factor in melanoma.

Furthermore, circRNAs have been shown to act as the sponges of miRNAs involved in melanoma progression. For example, circ_0082835, circ_0062270, circ_0079593, circ_0020710, circ_0025039, and circ_0002770 can respectively sponge miR‐429 [30], miR‐331‐3p [31], miR‐573 [33], miR‐370‐3p [26], miR‐198 [34] and miR‐331‐3p [35] to promote melanoma progression. Herein, we found circANKRD52 could bind to miR‐141‐3p, leading to upregulation of PRKACB, and downregulation of miR‐141‐3p restored melanoma cell growth, invasion, and angiogenic capabilities of HUVECs. Previous reports showed that miR‐141‐3p is identified to be decreased and reduces melanoma cell viability [36, 37]. PRKACB was selected as a functionally relevant target of miR‐141‐3p based on its Database‐predicted high expression in melanoma tissues and complete experimental validation by luciferase. Moreover, PRKACB blocks T‐cell chemokines to help melanoma escape immunity [38]. These findings suggested that circANKRD52 promoted the growth and invasion of melanoma cells by sponging miR‐141‐3p and upregulating PRKACB.

In conclusion, our findings demonstrated that circANKRD52 was upregulated in melanoma tissues and associated with poor prognosis in patients with melanoma. The circANKRD52 promoted the growth and invasion of melanoma cells by sponging miR‐141‐3p and upregulating PRKACB. Our studies might provide a novel therapeutic target for melanoma patients.

## Author Contributions

**Shaojun Chu:** data curation (lead). **Lingling Jia:** funding acquisition (equal). **Yulong Li:** data curation (equal). **Changjiang Zhao:** data curation (equal). **Yulin Sun:** data curation (equal). **Qin Zhou:** data curation (equal). **Dexiang Du:** formal analysis (equal). **Zihan Li:** methodology (equal). **Xin Huang:** funding acquisition (equal). **Hua Jiang:** funding acquisition (equal). **Baojin Wu:** funding acquisition (equal). **Yufei Li:** project administration (equal).

## Ethics Statement

Ethical approval was obtained from Shanghai East Hospital and conducted in accordance with the principles of the Declaration of Helsinki. The present study was approved by the Ethics Committee of Shanghai East Hospital (No. K‐KYSB‐2020‐0).

## Consent

The patients did not accept any therapy before the operation and signed written informed consent.

## Conflicts of Interest

The authors declare no conflicts of interest.

## Supporting information


**Figure S1:** (A, B) The transfections of oe‐circANKRD52 or sh‐circANKRD52 in A375 and SK‐MEL‐28 cells.

**Figure S2:** (A) The putative binding site between circANKRD52 and miR‐141‐3p. (B) bundant miR‐141‐3p was detected in the melanoma cells pulled down by circANKRD52‐WT.

**Figure S3:** (A, B) The transfections of miR‐141‐3p mimic or miR‐141‐3p inhibitor.

**Figure S4:** (A) The transfections of sh‐PRKACB or sh‐NC in the oe‐circANKRD52‐transfected A375 cells. (B) The transfections of oe‐PRKACB or sh‐NC in the sh‐circANKRD52‐transfected SK‐MEL‐28 cells.

## Data Availability

The datasets used during the current study are available from the corresponding author on reasonable request.
