# Supplementary figures and images for: CircANKRD52 Augments the Growth and Invasion of Melanoma Cells by Sponging miR‐141‐3p and Upregulating PRKACB
Source: J Cell Mol Med. 2025 Oct 31;29(21):e70909. doi: 10.1111/jcmm.70909 (PMC12578596; doi:10.1111/jcmm.70909)

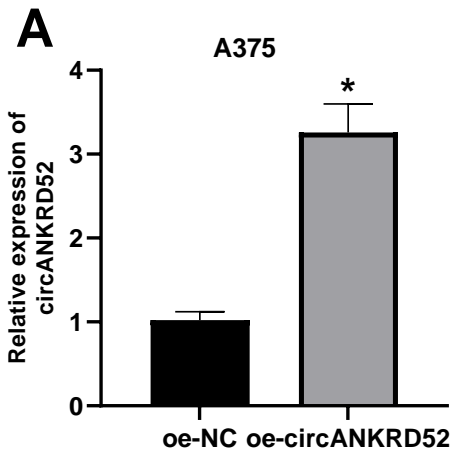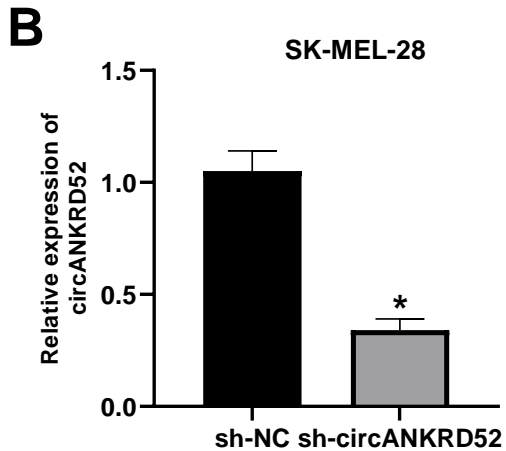

Supplement: Supplementary file 1 — Figure S1: (A, B) The transfections of oe‐circANKRD52 or sh‐circANKRD52 in A375 and SK‐MEL‐28 cells. [file JCMM-29-e70909-s003.pdf]

**A**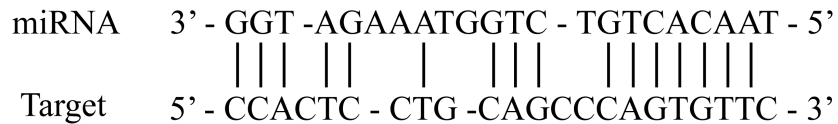**B**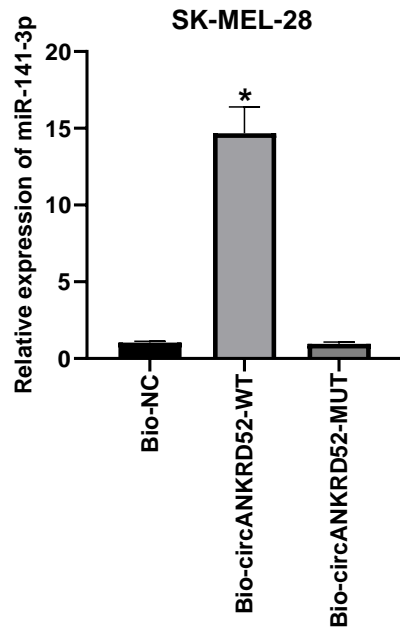

Supplement: Supplementary file 2 — Figure S2: (A) The putative binding site between circANKRD52 and miR‐141‐3p. (B) bundant miR‐141‐3p was detected in the melanoma cells pulled down by circANKRD52‐WT. [file JCMM-29-e70909-s001.pdf]

**A****A375**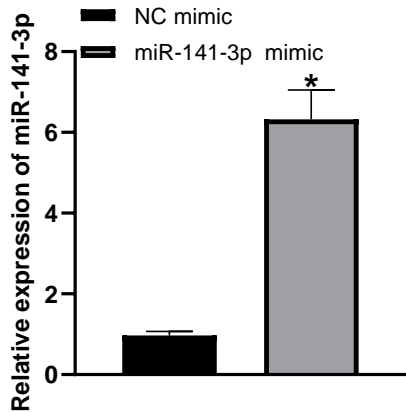**B****SK-MEL-28**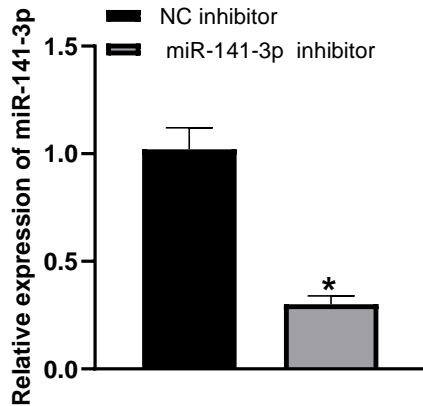

Supplement: Supplementary file 3 — Figure S3: (A, B) The transfections of miR‐141‐3p mimic or miR‐141‐3p inhibitor. [file JCMM-29-e70909-s002.pdf]

**A****A375**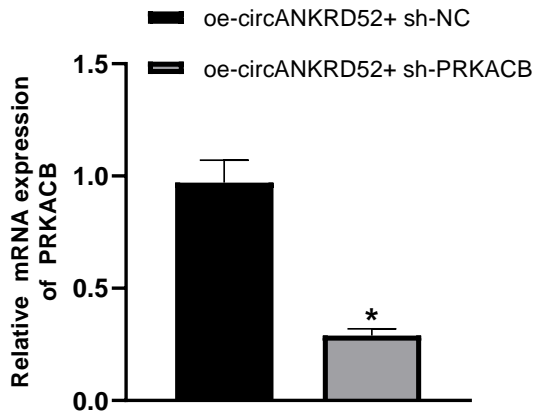**B****SK-MEL-28**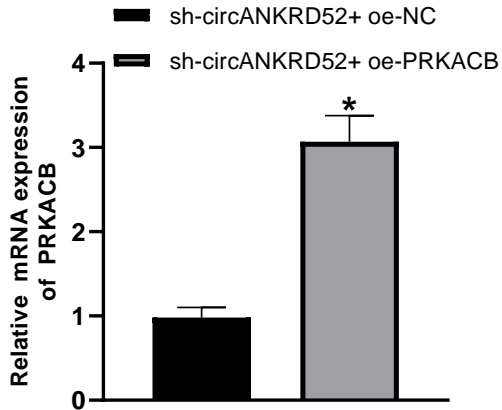

Supplement: Supplementary file 4 — Figure S4: (A) The transfections of sh‐PRKACB or sh‐NC in the oe‐circANKRD52‐transfected A375 cells. (B) The transfections of oe‐PRKACB or sh‐NC in the sh‐circANKRD52‐transfected SK‐MEL‐28 cells. [file JCMM-29-e70909-s004.pdf]
